# Regional inequalities in premature mortality in Great Britain

**DOI:** 10.1371/journal.pone.0193488

**Published:** 2018-02-28

**Authors:** Thomas Plümper, Denise Laroze, Eric Neumayer

**Affiliations:** 1 Department of Socioeconomics, Vienna University of Economics and Business, Vienna, Austria; 2 Centre for Experimental Social Sciences, Universidad de Santiago de Chile, Santiago, Chile; 3 Department of Geography & Environment, London School of Economics and Political Science (LSE), London, United Kingdom; Universidad del Desarrollo, CHILE

## Abstract

Premature mortality exhibits strong spatial patterns in Great Britain. Local authorities that are located further North and West, that are more distant from its political centre London and that are more urban tend to have a higher premature mortality rate. Premature mortality also tends to cluster among geographically contiguous and proximate local authorities. We develop a novel analytical research design that relies on spatial pattern recognition to demonstrate that an empirical model that contains only socio-economic variables can eliminate these spatial patterns almost entirely. We demonstrate that socioeconomic factors across local authority districts explain 81 percent of variation in female and 86 percent of variation in male premature mortality in 2012–14. As our findings suggest, policy-makers cannot hope that health policies alone suffice to significantly reduce inequalities in health. Rather, it requires strong efforts to reduce the inequalities in socio-economic factors, or living conditions for short, in order to overcome the spatial disparities in health, of which premature mortality is a clear indication.

## Introduction

The probability to die prematurely in Great Britain exhibits strong spatial patterns–stronger indeed than in most other Western European countries [1]. Premature mortality is higher up North–especially in Scotland–and the West of Great Britain, in former industrial centres of textiles, coal, and steel and in poorer and more urban areas [2]. The highest probability of premature death, defined here as dying before the age of 70, exists in and around Glasgow. In fact, this early industrial city in Scotland is infamous for the ‘Glasgow effect’ [3]: the extremely high premature mortality and associated low life expectancy of Glaswegians.

The National Institute for Health and Care Excellence estimates that in England alone around two thirds of deaths of those aged 75 or below (around 103,000 fatalities per year) are avoidable. The reason is that the most important direct causes, such as cancer, heart disease, stroke, respiratory and liver diseases “are preceded by long periods of ill-health mostly caused by lifestyle related factors” [4]. This estimate is of course contestable. Yet, another way of appreciating the substantial significance of preventable premature mortality derives from a counter-factual thought experiment: If all British local authority districts had the same low propensity for premature death as South Cambridgeshire, the total number of premature deaths would fall by approximately 33 percent–and some of the deaths in Cambridgeshire will be avoidable, too.

Research has pointed towards income as the main explanatory factor of health inequalities [5–8]. In Great Britain, mortality from liver diseases, respiratory diseases, cardiovascular diseases and cancer are on average two times more likely among the poorest quintile of the population than among the richest quintile of the population [9]. US data suggests that poor and less educated individuals are twice as likely as rich and well educated individuals to prematurely die from cancer [10]. In addition, mortality rates from cancer decline quicker among more affluent people [11]. But income and poverty are not the only potential culprits. Other factors that influence premature death, most of which correlate with income, include education [12–13], sectoral composition and professional occupation (blue collar versus white collar jobs) and social status [7, 14–18]. Though it tends to be inversely related with average income, premature mortality is also significantly higher in more densely populated areas [19–20].

This article uses a novel research design to analyse the extent to which particular types of spatial patterns in premature death are associated with spatial patterns in socioeconomic factors which create living conditions adverse to good health and which trigger behavioural traits that may cause diseases that eventually lead to an early death [21]. Specifically, we use a simple yet powerful technique we dub spatial pattern recognition. This technique estimates the strength of a spatial pattern in the residuals of an empirical estimation model that excludes relevant socioeconomic factors and compares these to the strength of spatial pattern that remains in the residuals once we estimate a different empirical model that is enriched by the socioeconomic factors of interest. We have chosen to analyse spatial patterns in terms of geo-coordinate location (the North-South and West-East divides), centrality, contiguity, proximity and urbanity.

Our analysis makes a second, substantive contribution. We draw attention to a range of specific spatial patterns in premature mortality, whereas the extant literature is either focused on the cross-sectional variation in premature mortality in general (without a specific focus on spatial patterns) or dominantly focuses exclusively on the North-South and West-East divides [22–30]. In addition, by analysing a larger set of socioeconomic factors we demonstrate that this more comprehensive set of determinants reduce the spatial patterns recognised to a much greater extent than existing studies find [14,22]. Specifically, in a cross-sectional analysis of premature mortality in 378 out of 380 local authorities in Great Britain in 2012–14, we find that the local authority characteristics of average income, dependency on welfare benefits, educational qualification, sectoral employment composition, socioeconomic position, and ethnic composition almost completely account for spatial patterns in premature mortality. In other words, the various types of spatial patterns in premature mortality are strongly reduced and sometimes entirely, or almost entirely, eliminated after the spatial patterns in socioeconomic factors has been taken into account.

## Spatial pattern recognition

Spatial patterns in outcomes result from two different causal processes: spatial clustering and spatial dependence. Spatial clustering occurs when independent variables are correlated across space. For example, most cultural variables, genetic features, many institutions are correlated across space and these correlations may bring about spatial patterns in outcomes, here the propensity to die prematurely. In contrast, spatially correlated outcomes may also exist because the units of analysis are not independent of each other, but are linked through interaction and exchange relations that trigger spatially dependent outcomes [23,24].

Spatial pattern recognition can be used to identify spatial patterns resulting from either causal process. Specifically, spatial pattern recognition proceeds in five major steps:

The first step estimates a stripped-down empirical baseline model that *excludes* the variables of theoretical interest–the variables which are supposed to generate the spatial pattern in the outcome variable. In the extreme, the baseline model is entirely empty though it could also include explanatory variables that are not of theoretical interest. The baseline model is used to compute the model’s residuals. The residuals represent the variation in the dependent or outcome variable not explained or accounted for by the explanatory variables. If the baseline model is entirely empty then the residuals are identical to the outcome variable.

The second step regresses the residuals from the stripped-down estimation model from step 1 on selected variables that identify specific spatial patterns in these residuals. This allows us to estimate the strength of specific types of spatial patterns. In our case, we employ this technique to estimate the strength of spatial patterns in premature mortality in Great Britain in five specific dimensions: geo-coordinate location, specifically the degree to which a district is located further North and further West on the British Isles (data taken from the UK Data Service Census Support); centrality, defined by geographical distance from the centre, here London; contiguity (defined as two districts being physically adjacent); proximity (defined as the inverse of Euclidean distance between the centroid of two districts); and urbanity, defined as population density of a local authority district (data taken from the British 2011 census). Of course, one could also estimate altitude, distance to border, and other spatially relevant variables where this makes sense. Spatial pattern recognition is a general technique that can employ any cardinally measured spatial dimension.

The third step re-estimates the model of stage 1 but this time *including* the variables of substantive theoretical interest. In our case, we include income and poverty, educational qualification, the sectoral composition of the economy, socioeconomic status and the ethnic composition of local authority districts into the estimation model. Like before, we compute the residuals of this empirically rich model. Since the number of regressors in the full model is larger than the number of regressors included in the benchmark model, the sum of squared residuals will decline and the R^2^ increases.

The fourth step repeats the second step but this time with the residuals of the empirically rich model estimated in step 3. We again arrive at an estimate of the strength of spatial patterns but this time with residuals derived from an estimation model that accounts for the theoretical variables of interest.

The fifth and final step compares the estimated strengths of spatial patterns identified in step 2 to the strengths of spatial patterns identified in step 4. Comparing the strengths of the spatial patterns of residuals left unexplained by the two models estimated in step 1 and step 3 provides an indicator of the decline in spatial patterns which result from the inclusion of theoretically interesting variables in the empirically rich model estimated in step 3. In other words, this final step of the spatial pattern recognition allows us to quantify to which extent the spatial patterns in the residuals has declined by adding the substantively interesting variables to the model. In our case, we obtain an estimate by how much the spatial structure in premature mortality declines by accounting for spatial structure in its socioeconomic determinants. Since the socioeconomic determinants vary strongly across local authorities and follow similar spatial patterns as premature mortality, the decline in spatial patterns in the premature mortality data caused by the inclusion of socioeconomic variables in step 3 is rather strong, as we will demonstrate in the next section.

## Data

We define premature mortality as the probability of dying before the age of 70, calculated on the basis of death tables for England, Wales and Scotland for the period 2012–14 taken from the Office of National Statistics for England and Wales and the National Records of Scotland. These life tables allow us to compare the survival rate of an artificial cohort of 100,000 individuals in each local authority based on observed, that is, actual age-dependent probabilities of dying. This in turn gives us a standardized propensity of premature mortality that is comparable across local authorities despite their differences in demographic composition (the age structure of their population), namely the number of individuals that do not reach the age of 70 in an artificial cohort of 100,000. For men, the mean of our dependent variable across local authorities is 18,790 (s.d. 3,498) with a range from 12,555 to 33,250; for women, mean premature mortality is 12,699 (s.d. 2,281) with a range from 8,445 to 21,412. Our findings are robust to employing instead the lower age threshold of 60 or the higher age threshold of 75 as the definition of premature mortality (detailed results shown in S1 and S2 Tables). We analyse premature mortality separately for men and women.

Our theoretically motivated explanatory factors are the socioeconomic determinants of premature mortality. The data for these variables come from the British 2011 census and from other statistics provided by the Office of National Statistics (ONS) and the Scotland Census in 2011. We use five sets of socioeconomic variables: income and poverty; education; sectoral composition of the economy; socioeconomic status; and ethnic composition. Specifically, we include mean district level income and the share of social welfare benefits claimants as a proxy for poverty. We use information on the highest level of educational qualification (broken down into 5 categories), on the types of economic sectors that provide employment (18 categories), on socioeconomic status composition (8 categories), and main ethnicities (5 categories). For comparability, these characteristics are measured as a proportion of the relevant local authority population (e.g. the percentage of the local population that has achieved a certificate of higher education and above). S3 and S4 Tables provides summary descriptive information for the variables.

Note that because we use exhaustive categories (leaving out one as reference category) for education, sectoral composition of the economy and socioeconomic status rather than continuous measures of these socioeconomic factors (e.g. years of schooling), we explicitly do not assume that the effect of, say, education on premature mortality is linear in the number of years of schooling which would be highly implausible. Allowing for further non-linear effects by including second degree polynomial terms of our explanatory variables results in only a small increase in goodness-of-fit with the data and leaves our substantive findings unchanged (results not reported here).

Given that the socioeconomic factors included in our estimation model are not mutually independent from each other and some might represent the causal mechanism by which others exert their effect, we do not evaluate the point estimates of individual variables or their statistical significance. All we are interested in here is the combined explanatory power that socioeconomic factors jointly exert on premature mortality. In future research, we will explore which of these socioeconomic factors are the key drivers.

The data provided by the ONS and the Scotland Census was complete for all 380 local authorities in Great Britain, with the exception of five missing observations for mean income. To address this issue we replaced the missing data with the mean of 100 imputed values. The imputations were carried out using a linear model that included the same socioeconomic variables used in the analysis of premature mortality (except mean income) plus population density. Results are almost identical if instead of multiple imputation we drop the five local authority districts with missing income data (results not reported here). Analyses were conducted using ordinary least squares regressions on premature mortality in 378 of the 380 local authorities in Great Britain. Estimation using a Poisson or Negative Binomial model gives substantively identical results (results not reported here). Following [31], we exclude data for the City of London and the Isle of Scilly because their low population sizes render premature mortality figures unreliable. Results are practically identical if we weight observations by population size (see S5 Table).

One potential concern is whether the spatial patterns recognised by our analysis are driven by outliers and are thus not representative for the sample. In a robustness test, we show that results are practically identical if we exclude outliers from the spatial recognition analysis, employing the outlier definition of [32]–see S6 Table for results.

## Analyses

**Fig 1:**
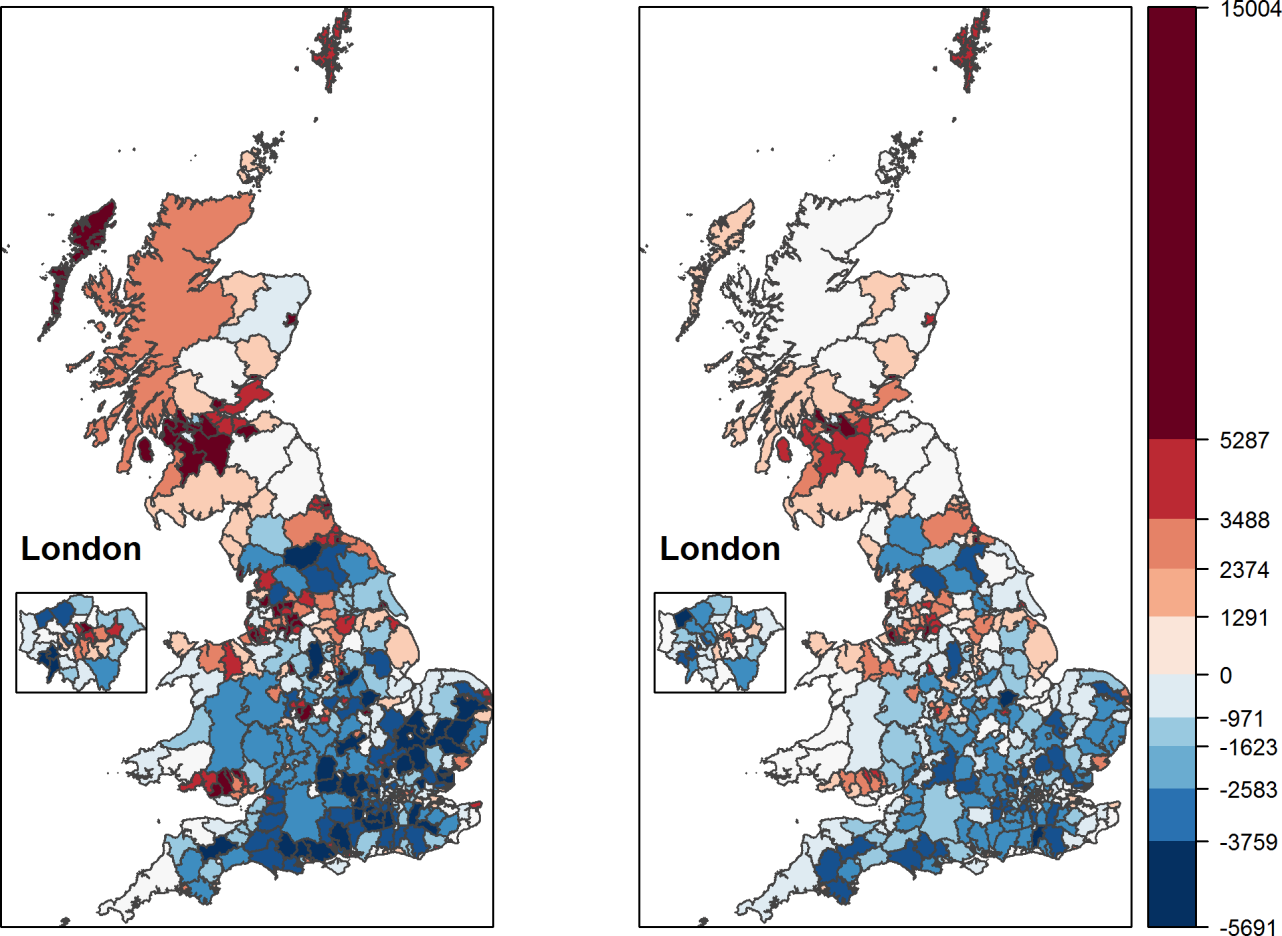
Above and below median premature mortality of men (left) and women (right) in Great Britain.

We estimate an entirely empty model in step 1. Accordingly, the ‘residuals’ are simply equal to the outcome variable, premature mortality rates. Fig 1 shows the spatial pattern in premature mortality data. The maps reveal increasing degrees of above median (increasingly darker red) and below median (increasingly darker blue) premature mortality rates for men on the left and women on the right. For ease of comparison, both maps use the same scale: deciles of male premature mortality.

It becomes evident that the odds of dying prematurely are much higher in the North-West with the highest premature mortality rates occurring in and around Glasgow, while the lowest rates occur in a belt that stretches from East Anglia to Dorset. In addition, old industrial areas such as Birmingham, Liverpool, Manchester, parts of East London (particularly for men), and North and South Wales have higher premature mortality rates. So do urban areas compared to surrounding, more rural, neighbourhoods. Even Aberdeen and Dundee stand out from the surrounding areas, and so do Bristol, Hull and Grimsby.

In step 2, we regress premature mortality on specified variables that can identify specific spatial patterns in premature mortality. Recall that this second step identifies patterns in specified spatial dimensions in the outcome variable of interest, premature mortality. The first two rows present results from regressing male and female premature mortality on the geo-coordinate location of local authorities. The next row reports results from a separate regression on geographical distance from the centre. The next two rows are based on separate estimations using, respectively, average premature mortality in geographically contiguous and proximate districts. Finally, the last row reports results from regressing premature mortality on the population density of a local authority. S1 Appendix provides summary descriptive information for the variables we employ for the spatial pattern recognition.

Table 1 confirms quantitatively what can already be gleaned from the maps: spatial patterns tend to be stronger for men than for women. For Great Britain as a whole as well as for England, all five spatial patterns are strong and statistically significant, with the exception of urbanity for female premature mortality. The geo-coordinate variables of ‘Northness’ and ‘Westness’ capture the well-known North-South and West-East divides in premature mortality in Great Britain. Of course, for a country like Great Britain, where the unit with the largest number or the strongest links to all other units–typically though not always the capital–is located in the very South-East, geo-coordinate location and centrality capture similar structures. We also find evidence for spatial patterns in terms of contiguity and proximity. Lastly, we find evidence for higher premature mortality rates in cities compared to more rural areas for men and women in Great Britain, England, and Scotland. In fact, within Scotland urbanity is the only spatial pattern recognised by our analysis.

**Table 1:** Spatial patterns in observed premature mortality rates (residuals of empty estimation model).

	Great Britain		England		Scotland	
	male	female	male	female	male	female
Northness	0.0078**	0.0057**	0.0102**	0.0079**	-0.0007	-0.0033
	(0.0010)	(0.0007)	(0.0013)	(0.0009)	(0.0038)	(0.0033)
Westness	0.0067**	0.0042**	0.0036*	0.0020	0.0142	0.0014
	(0.0016)	(0.0011)	(0.0018)	(0.0012)	(0.0077)	(0.0054)
Centrality	0.0105**	0.0073**	0.0119**	0.0086**	-0.0017	-0.0034
	(0.0011)	(0.0008)	(0.0016)	(0.0010)	(0.0041)	(0.0033)
Contiguity	0.7224**	0.7650**	0.6920**	0.7158**	-0.0736	0.1332
	(0.0755)	(0.0681)	(0.0740)	(0.0720)	(0.3516)	(0.3812)
Proximity	1.9100**	1.9156**	1.5749**	1.4032**	1.6734	2.8745
	(0.4204)	(0.4157)	(0.4198)	(0.4082)	(1.9262)	(1.7114)
Urbanity	22.3832*	5.3561	28.1430**	9.4712	299.68**	169.28**
	(9.5638)	(5.1169)	(10.7731)	(5.8908)	(49.1718)	(36.2001)

Note: Robust standard errors in parentheses.

**, * statistically significant at .01, .05 level.

The regression model in which we regress premature mortality on its socioeconomic determinants takes us to step 3 of the spatial pattern recognition exercise. The empirical estimation model now includes a rich battery of socioeconomic variables. For expositional reasons only, we express the dependent variable as an actual percentage (rather than as the number of survivors out of a hypothetical population of 100,000). Naturally, this does not change any of the substance of the estimations. Results presented in Table 2 show that these socioeconomic characteristics of local authorities account for between 86 percent (men) and 81 percent (women) of the cross-sectional variation in premature mortality in Great Britain. This very substantial overall explanatory power indicates the importance of inequality across local authorities in socioeconomic factors for explaining inequality across local authorities in premature mortality. As we have discussed before, because the variables are not mutually independent from each other, it is not possible to interpret the estimated coefficients of single variables as their isolated effect. For our research interest, only the joint explanatory power of the set of socioeconomic factors matters.

**Table 2:** The socioeconomic empirical model of premature mortality.

	male	female
Mean income	-0.0002	0.0001
	(0.0002)	(0.0001)
Benefit claimants	0.0055	0.0050
	(0.0015)	(0.0012)
Highest educational qualification composition		
GCSE (grades D-G)	0.1989	0.1319
	(0.0494)	(0.0384)
GCSE (grades A-C)	-0.0097	-0.0016
	(0.1001)	(0.0840)
A level	-0.0996	-0.1119
	(0.1052)	(0.0793)
Certificate of higher education and above	0.1700	0.0697
	(0.0712)	(0.0573)
Employment by economic sector composition		
Agriculture	-0.2236	0.0524
	(0.1562)	(0.0971)
Mining	0.0086	-0.0181
	(0.1709)	(0.1107)
Manufacturing	0.1063	0.1131
	(0.0969)	(0.0601)
Gas & Electricity	0.0031	0.0392
	(0.2157)	(0.1601)
Water	0.3241	0.1579
	(0.3630)	(0.2693)
Construction	-0.2709	-0.1544
	(0.1395)	(0.0848)
Retail	-0.0763	0.0000
	(0.1021)	(0.0698)
Transport	0.1155	0.0772
	(0.1066)	(0.0604)
Hospitality	0.2414	0.1079
	(0.1389)	(0.0948)
Information Technology	0.3237	0.1728
	(0.1193)	(0.0697)
Finance	0.1470	0.0328
	(0.0986)	(0.0575)
Real estate	0.1277	0.2720
	(0.3794)	(0.2480)
Academic/Science	0.3862	0.2212
	(0.1368)	(0.0902)
Administration	0.3721	0.1436
	(0.1683)	(0.1243)
Public Administration	0.0627	0.0641
	(0.1003)	(0.0611)
Education	-0.0811	-0.0815
	(0.1085)	(0.0713)
Health	0.3903	0.1869
	(0.1067)	(0.0663)
Socioeconomic status composition		
Higher managerial	-0.9617	-0.5427
	(0.1445)	(0.1000)
Lower managerial	-0.2620	-0.2952
	(0.1134)	(0.0911)
Intermediate occupations	-0.1730	-0.0798
	(0.1102)	(0.0872)
Small employers	-0.1776	-0.2217
	(0.1284)	(0.0879)
Lower supervisory	-0.1713	0.0197
	(0.2104)	(0.1575)
Semi-routine occupations	-0.4342	-0.3803
	(0.1196)	(0.1018)
Routine occupations	0.1398	-0.0880
	(0.1114)	(0.0803)
Ethnic composition		
Mixed	0.3218	0.4318
	(0.2536)	(0.1854)
Asian	0.3291	0.3661
	(0.1763)	(0.1204)
Black	0.2323	0.3207
	(0.1806)	(0.1262)
White	0.3686	0.4027
	(0.1758)	(0.1211)
Constant	-0.0926	-0.1598
	(0.1736)	(0.1132)
Observations	378	378
Adjusted R-squared	0.86	0.81

Note: Robust standard errors in parentheses. Omitted reference categories are ‘not working’, ‘other ethnicity’, ‘entry level educational qualification’ and ‘other sector’, respectively.

A model that explains premature mortality to a very large extent does not necessarily explain the spatial patterns in premature mortality to the same extent. Our model does, however, as the third, final and most important step of the spatial pattern recognition exercise demonstrates.

Step 4 of our analysis repeats the regressions conducted with the residuals from the empty model of step 2 but this time with the residuals from the empirically rich ‘socioeconomic model’.

Finally, step 5 analyses the extent to which the battery of socioeconomic factors have reduced or even eliminated the spatial patterns in premature mortality. Table 3 compares the estimates of the strength of correlation in the five spatial dimensions in the observed values of premature mortality, as previously reported in Table 1 above, to the strength of correlation in the same spatial dimensions but this time in the residuals from the socioeconomic empirical model, as reported in Table 4. For simplicity, results are shown for both men and women for Great Britain as a whole only.

**Table 3:** Reduction in strength of spatial patterns in observed premature mortality versus spatial patterns in residuals from the socioeconomic empirical model.

	Observed	Observed	Residuals	Residuals	Decline	Decline
	male	Female	male	Female	male	female
Northness	0.0078**	0.0057*	0.0005	0.0005	93.6%	91.2%
	(0.0010)	(0.0007)	(0.0005)	(0.0004)		
Westness	0.0067**	0.0042*	0.0013	0.0007	80.6%	83.3%
	(0.0016)	(0.0011)	(0.0007)	(0.0005)		
Centrality	0.0105**	0.0073*	0.0009	0.0006	91.4%	91.8%
	(0.0011)	(0.0008)	(0.0005)	(0.0004)		
Contiguity	0.7224**	0.7650**	0.0404	0.0894**	94.4%	88.3%
	(0.0755)	(0.0681)	(0.0306)	(0.0315)		
Proximity	1.9100**	1.9156**	-0.0562	0.0749	102.9%	96.1%
	(0.4204)	(0.4157)	(0.1510)	(0.1623)		
Urbanity	22.3832*	5.3561	2.6274	1.0377	88.3%	80.6%
	(9.5638)	(5.1169)	(2.4780)	(1.7396)		

Note: Robust standard errors in parentheses.

**, * statistically significant at .01, .05 level.

**Table 4:** spatial patterns in residuals from the socioeconomic empirical model.

	Great Britain		England		Scotland	
	male	female	male	female	male	female
Northness	0.0005	0.0005	0.0006	0.0008	0.0073**	0.0029
	(0.0005)	(0.0004)	(0.0006)	(0.0005)	(0.0017)	(0.0021)
Westness	0.0013	0.0007	0.0021**	0.0010	0.0079*	-0.0007
	(0.0007)	(0.0005)	(0.0007)	(0.0006)	(0.0036)	(0.0032)
Centrality	0.0009	0.0006	0.0012	0.0008	0.0071**	0.0033
	(0.0005)	(0.0004)	(0.0007)	(0.0005)	(0.0018)	(0.0023)
Contiguity	0.0404	0.0894**	0.0743*	0.1179**	0.0201	0.2307
	(0.0306)	(0.0315)	(0.0336)	(0.0382)	(0.1251)	(0.1634)
Proximity	-0.0562	0.0749	-0.1198	-0.1645	0.2368	1.3679
	(0.1510)	(0.1623)	(0.1484)	(0.1592)	(0.8613)	(0.7898)
Urbanity	2.6274	1.0377	2.2847	1.2113	100.0265	53.8693
	(2.4780)	(1.7396)	(2.5268)	(1.7341)	(58.3692)	(38.6448)

Note: Robust standard errors in parentheses.

**, * statistically significant at .01, .05 level.

With the exception of the West-East divide, we find that socioeconomic factors reduce the spatial patterns in premature mortality more for men than for women. However, these gender differences are much less pronounced than the overall very strong decline in spatial patterns for both men and women and across all five spatial dimensions. After controlling for socioeconomic factors, the North-South and West-East divide decline by between 80.6 and 93.6 percent. The divides are not entirely eliminated. The coefficients of the geographical location remain positive, even if much reduced in size. Not surprisingly, given London’s location in the South-East of Great Britain, the centrality pattern declines similarly strongly as the geo-coordinate location pattern. The contiguity spatial pattern is similarly strongly reduced by accounting for socioeconomic factors, while the proximity spatial pattern is entirely eliminated for men and almost entirely eliminated for women. The spatial pattern of higher mortality in more densely populated urban areas is reduced by between 80.6 and 88.3 percent.

**Fig 2:**
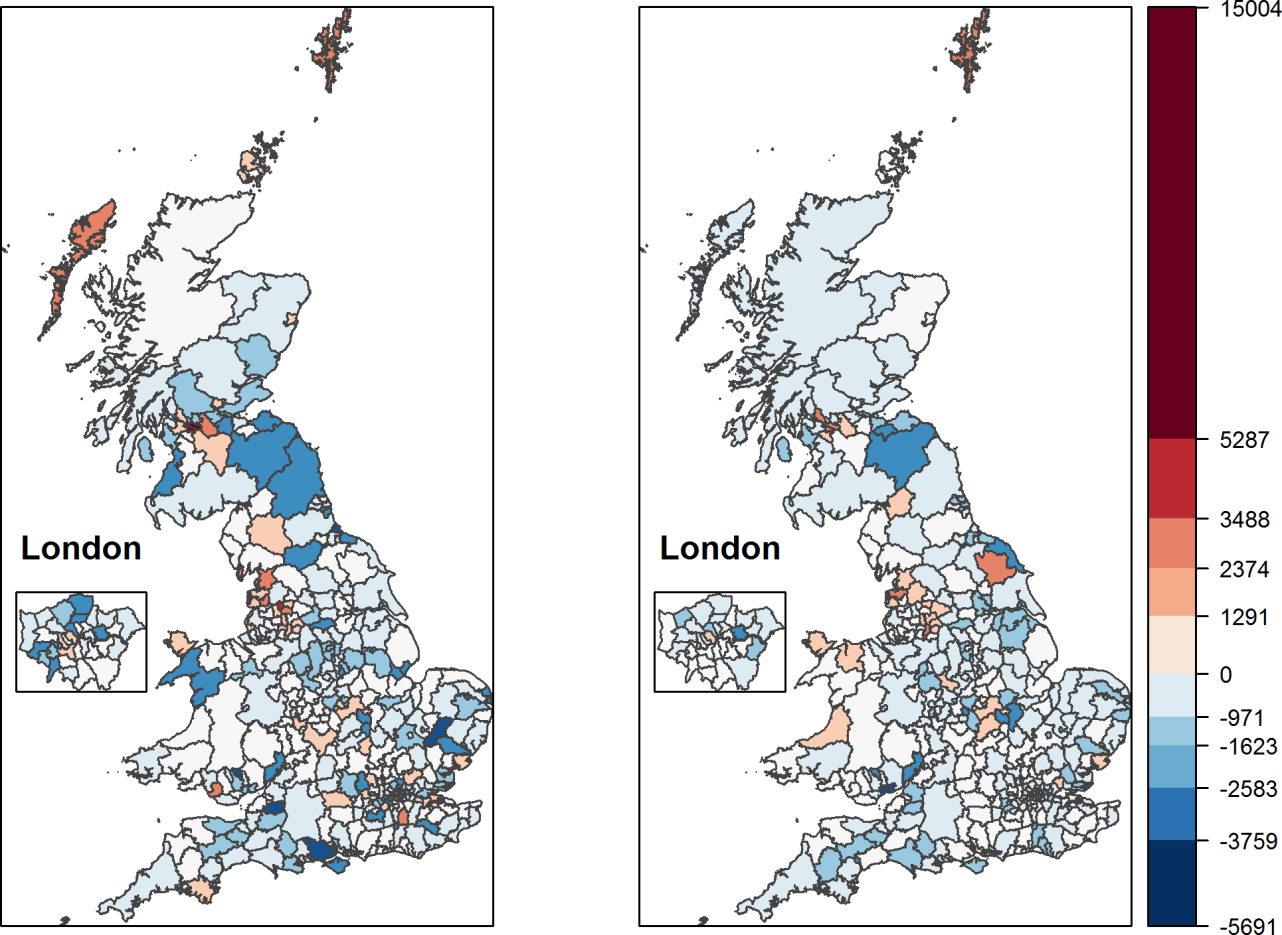
Unexplained variation in premature mortality for men (left) and women (right).

We can also visualize the strong explanatory power of socioeconomic factors in terms of accounting for the spatial patterns in premature mortality with the help of maps again. Fig 2 visualizes, separately for men and women, the residuals from our estimation model, that is, the variation in premature mortality unexplained by the socioeconomic explanatory variables. Even a superficial comparison of Figs 1 and 2 reveals that the spatial patterns in premature mortality of men and women, which were so prominent in Fig 1, are strongly reduced by the socioeconomic factors included in our model. Yet, minor spatial patterns survive. Pockets of unexplained excess premature mortality for men and women continue to exist in and around Glasgow and Manchester, whereas lower than expected premature mortality is rather unsystematically distributed. Interestingly, for London our model tends to over-predict actual mortality rates. This is consistent with previous findings of a positive ‘London effect’, with mortality lower than expected based on socioeconomic factors, for which immigration of relatively healthy people might be the explanation [33].

## Discussion

Socioeconomic factors explain variation in premature mortality across local authorities in Great Britain to a very large extent. More importantly, taking into account spatial variation in socioeconomic factors strongly reduces and sometimes fully eliminates the spatial patterns that one can find in premature mortality across the British Isles in terms of geo-coordinate location (the North-South and West-East divides), centrality, contiguity, proximity and urbanity. This holds true for both men and women: we found some but very minor gender differences. This finding of our analysis, which is based on a larger set of socioeconomic factors than employed in previous studies, stands in marked contrast to [24] whose “most striking conclusion” on p. 906f. is “that, even after taking into account levels of social deprivation and area type, marked regional differences in all-cause mortality rates are still apparent.” In other words, we demonstrate that a broader set of socioeconomic determinants than used hitherto in the extant literature can explain spatial patterns in (premature) mortality to a much greater extent than was known before.

Socioeconomic factors should be understood as root causes of premature mortality, not as direct causes. Take income as an example. In a narrow view, income has no direct causal effect on health: if we make 1,000 poor individuals significantly richer, but force them not to change their behaviour and not to spend the additional income, our treatment presumably has little or no influence on health. If, however, we increase the income of individuals without imposing such constraints on them, then individuals that become richer are likely to change their behaviour: they may move house, start purchasing healthier food, reduce alcohol consumption, buy additional education, spend more on health care, and so on. In other words, income influences lifestyle choices, and lifestyles affect health in the long run. In our terminology: income is a root cause, but not the causal mechanism for good health, with the relation between these two factors also far from being perfectly understood: the estimated effect of income on health declines by roughly 25 percent after controlling for risk factors or for employment status [8].

Like income, education does not directly prevent premature mortality. However, it indirectly influences premature mortality through its influence on professional choice, income, nutrition, smoking habits and so on. Better educated people are healthier on average not because they are better educated, but because on average better educated people lead healthier lives. Sectoral composition can directly influence premature deaths through industry-specific risks and accidents. Sector-specific employment may also indirectly affect health. For example, working in shifts, which is much more common in some sectors than in others, has been associated with a significantly higher propensity for coronary heart diseases [34–35]. Sectoral composition and socioeconomic professional status also influence lifestyle choices.

While socioeconomic factors strongly reduce the spatial patterns in premature mortality they do not fully eliminate them. For example and perhaps most importantly, Glasgow’s high premature mortality rate remains an outlier in our analysis. It tops the list of unexplained excess premature mortality for both men and women in Great Britain. Glaswegian men are about 14.5 and Glaswegian women about 8.7 percentage points more likely to die prematurely than, respectively, men and women in the average local authority. Our model reduces the excess probability of premature mortality that is not caused by socioeconomic factors for Glaswegian men to 5.6 percent and for women to 3.3 percent. These figures may be considered a substantial reduction, but almost 40 percent of the premature mortality in Glasgow remains unexplained by our model.

A limitation of our study, which is focused on Great Britain, is that it is unclear to what extent our central finding–that accounting for socioeconomic factors very strongly reduces and sometimes eliminates spatial patterns in premature mortality–holds for other countries. Our study for Germany demonstrates that the finding is also valid for this country but the external validity beyond that needs to be demonstrated in future research [36].

There are a couple of potential objections critics might raise against socioeconomic factors as root causes of premature mortality and against our results providing supporting evidence in this regard. As the first potential objection, other causes of premature mortality can also spatially cluster and if they are the true causes of the spatial patterns in premature mortality then our estimation results are spurious. We can think of three different causes of premature mortality that spatially cluster: genetic variation, climatic conditions and health care facilities and expenditures. As concerns genetic variation, the British Isles were populated by four different gen pools: early immigration was dominated by Saxons in the South-East, the Britons in the South-West, the Picts in the North-East and the Irish in the North-West. However, the effect of this genetic variation on the propensity for various diseases is weak [37]. It is therefore highly unlikely that genetic dispositions explain the large variation in premature mortality in Great Britain, though the current genetic distribution still resembles to some extent the migration routes into the British Isles. Climatic conditions do influence mortality and the North and the West receive much more precipitation and enjoy fewer hours of sunshine than the South-East of Great Britain. The North is also colder than the South. However, research demonstrates that differences in climatic conditions have little influence on mortality–only unusual extreme weather, i.e. strong deviation from long-term climatic conditions, exerts a strong influence on mortality [38]. Health care facilities and health expenditures might also spatially cluster. We were not able to get reliable information on potential spatial patterns in the care provision and health expenditures by the National Health Service. However, if anything, such spatial clustering would tend to mitigate against the spatial patterns in premature mortality that we have identified in the previous section. For example, access to health care is typically easier in more urban than in more rural areas. Health expenditures are likely to be tilted toward pockets of excessive premature mortality rather than against them.

Moving to the second potential objection, instead of adverse socioeconomic factors causing premature mortality, poor health, which promotes premature mortality, can have adverse socioeconomic impacts. For example, individuals that are prone to regularly fall ill may be less successful in investing in education, may hold jobs of lower socioeconomic status and be less able to put outstanding effort into work and therefore receive lower income. Reverse causality can occur at the individual level to some extent but it cannot explain the strong spatial patterns across local authorities. There is no plausible exogenous factor that lets some local authorities miraculously have good health and other local authorities have bad health, which then causes favourable socioeconomic conditions in the former and adverse socioeconomic conditions in the latter. We thus flatly dismiss reverse causality as a credible objection.

## Conclusion

In a world in which all individuals were identical and had identical living conditions, premature mortality would not systematically vary across space. Though random processes would cause some variation in premature mortality of individuals and in premature mortality rates at local authority levels, these variations would be unlikely to form spatial patterns. In the real world in which individuals are not identical and living conditions vary strongly, health outcomes including premature mortality show clear spatial patterns.

Recently, the Westminster government placed health inequalities across Great Britain high on its political agenda. Then Health Secretary Jeremy Hunt called the scale and scope of regional differences in premature mortality shocking and concluded that “this (…) variation in early and unnecessary deaths means people's lives are needlessly cut short, and that cannot continue unchecked.” For once, the opposition agreed. Representatives of the Labour Party called for a “one nation approach” to end health inequalities [39].

Our findings are relevant to the Department of Health’s plan to reduce or even eliminate health inequalities. The Department of Health suggests that by providing “local areas with information to help them understand their own position” and by targeting “specific health challenges”, the regional disparities in premature death can be eliminated [39]. This is unlikely to succeed. Targeted health spending can prolong the lives of those who develop life-threatening conditions and thus mitigate regional disparities in premature mortality. But unless governments tackle the root causes of premature mortality–the socioeconomic factors that create adverse living conditions and influence behavioural traits that result in unhealthy lifestyle–they will merely mitigate the symptoms. Increased health spending cannot eliminate spatial patterns in premature mortality *after* individuals have acquired diseases that are very likely to eventually kill them. For example, 97 percent of patients with gallbladder cancer die within 5 years after the diagnosis. Other types of cancer–pancreatic cancer, liver cancer, lung cancer, oesophageal cancer–also have mortality rates above 80 percent.

Our research suggests that the goal of eliminating health inequalities can only be achieved if governments tackle and reduce socioeconomic inequalities that matter: inequalities in income, education and other socioeconomic factors. Accordingly, we believe that a consistent combination of economic, social and education policies need to complement and underpin better and more targeted health services if governments are serious about tackling health inequalities. That UK governments have failed to fulfil their self-proclaimed targets of reducing, over the period 2001 to 2010, by 10 per cent the gap in life expectancy between the bottom quintile of local authorities and the population as a whole is telling and supports our interpretation [40–41]. Of course, tackling the geographical disparities in living conditions represents a huge task to policy makers, but without it the chances to reach the political goal of equal health conditions across local authorities are slim.

## Supporting information

S1 Appendix (DOCX)

S1 Table Results for lower age threshold of 60 of Table 4 Reduction in Strength of Spatial Patterns in Observed Premature Mortality Versus Spatial Patterns in Residuals from the Socioeconomic Empirical Model. (DOCX)

S2 Table Results for higher age threshold of 75 of Table 4. Reduction in Strength of Spatial Patterns in Observed Premature Mortality Versus Spatial Patterns in Residuals from the Socioeconomic Empirical Model.
(DOCX)

S3 Table Summary of descriptive variable information. Variables employed in spatial pattern recognition (for Great Britain) (Tables 1 and 3 in text).
(DOCX)

S4 Table Summary of descriptive variable information. Variables employed in regressing premature mortality on its socioeconomic determinants (Table 2).
(DOCX)

S5 Table Results for observations weighted by population size of Table 4. Reduction in Strength of Spatial Patterns in Observed Premature Mortality Versus Spatial Patterns in Residuals from the Socioeconomic Empirical Model.
(DOCX)

S6 Table Results from outlier analysis of Table 4. Reduction in Strength of Spatial Patterns in Observed Premature Mortality Versus Spatial Patterns in Residuals from the Socioeconomic Empirical Model.
(DOCX)
