# Supplementary material for: Regional inequalities in premature mortality in Great Britain
Source: PLoS One. 2018 Feb 28;13(2):e0193488. doi: 10.1371/journal.pone.0193488 (PMC5831001; doi:10.1371/journal.pone.0193488)
Supplement: S1 Appendix — (DOCX) [file pone.0193488.s001.docx]

**Appendix**

**Summary descriptive variable information**

1. Variables employed in spatial pattern recognition (for Great Britain) (tables 1 and 3)

| Variable | N | mean | s.d. | min | max |
| --- | --- | --- | --- | --- | --- |
| Male premature mortality rate | 378 | 18790.26 | 3498.20 | 12555.0 | 33250.36 |
| Female premature mortality rate | 378 | 12699.00 | 2280.96 | 8445.00 | 21412.44 |
| Northness | 378 | 303858.1 | 176933.7 | 54268.21 | 1168120 |
| Westness | 378 | -436517 | 101156.7 | -647805 | -111367 |
| Centrality | 378 | 197654.7 | 163328.7 | 3986.234 | 991106 |
| Contiguity (male) | 373 | 18114.08 | 2543.154 | 12555 | 27422.52 |
| Contiguity (female) | 373 | 12306.95 | 1756.166 | 7639 | 18891.13 |
| Proximity (male) | 378 | 18815.57 | 452.5231 | 18072.55 | 20138.17 |
| Proximity (female) | 378 | 18815.57 | 452.5231 | 18072.55 | 20138.17 |
| Urbanity | 378 | 15.58807 | 29.26909 | 0.09 | 321.1 |

Note: 5 local authorities are not contiguous to any other authorities and therefore have missing values for the contiguity variable.

1. Variables employed in regressing premature mortality on its socioeconomic determinants (table 2)

| Variable | | N | | mean | s.d. | | min | max | |
| --- | --- | --- | --- | --- | --- | --- | --- | --- | --- |
| Male premature mortality rate | | 378 | | 0.188 | 0.035 | | 0.125 | 0.333 | |
| Female premature mortality rate | | 378 | | 0.127 | 0.023 | | 0.845 | 0.214 | |
| Mean income | | 378 | | 33572.15 | 8599.59 | | 22338.00 | 119645.00 | |
| Benefit claimants | | 378 | | 2.634 | 1.291 | | 0.800 | 7.100 | |
| Highest educational qualification composition | |  | | |  | | |  |  |
| GCSE (grades D-G) | | 378 | | 0.144 | 0.035 | | 0.058 | 0.283 | |
| GCSE (grades A-C) | | 378 | | 0.156 | 0.019 | | 0.078 | 0.186 | |
| A level | | 378 | | 0.120 | 0.017 | | 0.076 | 0.192 | |
| Certificate of higher education and above | | 378 | | 0.268 | 0.074 | | 0.142 | 0.536 | |
| Employment by economic sector composition | |  | | |  | | |  |  |
| Agriculture | | 378 | | 0.012 | 0.016 | | 0.000 | 0.100 | |
| Mining | | 378 | | 0.003 | 0.007 | | 0.000 | 0.089 | |
| Manufacturing | | 378 | | 0.094 | 0.039 | | 0.018 | 0.237 | |
| Gas & Electricity | | 378 | | 0.006 | 0.004 | | 0.001 | 0.039 | |
| Water | | 378 | | 0.007 | 0.003 | | 0.001 | 0.031 | |
| Construction | | 378 | | 0.080 | 0.015 | | 0.029 | 0.125 | |
| Retail | | 378 | | 0.160 | 0.021 | | 0.095 | 0.235 | |
| Transport | | 378 | | 0.049 | 0.017 | | 0.024 | 0.155 | |
| Hospitality | | 378 | | 0.056 | 0.017 | | 0.032 | 0.145 | |
| Information Technology | | 378 | | 0.036 | 0.021 | | 0.010 | 0.126 | |
| Finance | | 378 | | 0.040 | 0.025 | | 0.007 | 0.215 | |
| Real estate | | 378 | | 0.014 | 0.004 | | 0.006 | 0.034 | |
| Academic/Science | | 378 | | 0.062 | 0.027 | | 0.020 | 0.192 | |
| Administration | | 378 | | 0.047 | 0.009 | | 0.027 | 0.088 | |
| Public Administration | | 378 | | 0.062 | 0.021 | | 0.023 | 0.260 | |
| Education | | 378 | | 0.097 | 0.018 | | 0.059 | 0.236 | |
| Health | | 378 | | 0.127 | 0.023 | | 0.067 | 0.191 | |
| Socioeconomic status composition | |  | | |  | | |  |  |
| Higher managerial | | 378 | | 0.099 | 0.035 | | 0.039 | 0.215 | |
| Lower managerial | | 378 | | 0.210 | 0.032 | | 0.128 | 0.320 | |
| Intermediate occupations | | 378 | | 0.132 | 0.019 | | 0.079 | 0.196 | |
| Small employers | | 378 | | 0.098 | 0.026 | | 0.049 | 0.185 | |
| Lower supervisory | | 378 | | 0.075 | 0.015 | | 0.030 | 0.117 | |
| Semi-routine occupations | | 378 | | 0.146 | 0.027 | | 0.069 | 0.220 | |
| Routine occupations | | 378 | | 0.117 | 0.034 | | 0.043 | 0.229 | |
| Ethnic composition | |  | | |  | | |  |  |
| Mixed | | 378 | | 0.017 | 0.014 | | 0.002 | 0.076 | |
| Asian | | 378 | | 0.052 | 0.073 | | 0.004 | 0.435 | |
| Black | | 378 | | 0.021 | 0.042 | | 0.000 | 0.272 | |
| White | | 378 | | 0.904 | 0.123 | | 0.290 | 0.993 | |

Note: in table 2, premature mortality is expressed as a percentage.

|  |  |  |  |  |  |
| --- | --- | --- | --- | --- | --- |

**Results from outlier analysis**

*Table 4: Reduction in Strength of Spatial Patterns in Observed Premature Mortality Versus Spatial Patterns in Residuals from the Socioeconomic Empirical Model*

|  | Observed | Observedd | Residuals | Residuals | Decline | Decline |
| --- | --- | --- | --- | --- | --- | --- |
|  | male | Female | male | female | male | female |
| Northness | 0.0091** | 0.0064** | 0.0001 | 0.0000 | 98.9% | 100.0% |
|  | (0.0009) | (0.0006) | (0.0005) | (0.0004) |  |  |
| Westness | 0.0053** | 0.0044** | 0.0014* | 0.0011* | 73.6% | 75.0% |
|  | (0.0014) | (0.0009) | (0.0007) | (0.0005) |  |  |
| Centrality | 0.0119** | 0.0082** | 0.0005 | 0.0002 | 95.8% | 97.6% |
|  | (0.0009) | (0.0006) | (0.0006) | (0.0004) |  |  |
| Contiguity | 0.7772** | 0.8344** | 0.0215 | 0.0589 | 97.2% | 92.9% |
|  | (0.0546) | (0.0462) | (0.0325) | (0.0315) |  |  |
| Proximity | 1.8575** | 1.5939** | -0.2066 | -0.0669 | 111.1% | 104.2% |
|  | (0.3422) | (0.3335) | (0.1485) | (0.1619) |  |  |
| Urbanity | 42.4830** | 14.8333** | 0.4785 | -1.5115 | 98.9% | 110.2% |
|  | (7.3797) | (4.6325) | (2.6025) | (1.9408) |  |  |

Note: Robust standard errors in parentheses. **, * statistically significant at .01, .05 level.

**Results for lower age threshold of 60**

*Table 4: Reduction in Strength of Spatial Patterns in Observed Premature Mortality Versus Spatial Patterns in Residuals from the Socioeconomic Empirical Model*

|  | Observed | Observed | Residuals | Residuals | Decline | Decline |
| --- | --- | --- | --- | --- | --- | --- |
|  | male | Female | male | female | male | female |
| Northness | 0.0043** | 0.0026** | 0.0003 | 0.0002 | 93.0% | 92.3% |
|  | (0.0005) | (0.0003) | (0.0003) | (0.0002) |  |  |
| Westness | 0.0048** | 0.0023** | 0.0007 | 0.0002 | 85.4% | 91.3% |
|  | (0.0009) | (0.0005) | (0.0004) | (0.0003) |  |  |
| Centrality | 0.0065** | 0.0036** | 0.0006 | 0.0003 | 90.8% | 91.7% |
|  | (0.0006) | (0.0003) | (0.0003) | (0.0002) |  |  |
| Contiguity | 0.7316** | 0.7221** | 0.0207 | 0.0360 | 97.2% | 95.0% |
|  | (0.0805) | (0.0722) | (0.0345) | (0.0383) |  |  |
| Proximity | 2.0885** | 1.9590** | 0.0206 | 0.1480 | 99.0% | 92.4% |
|  | (0.4501) | (0.4179) | (0.2024) | (0.2011) |  |  |
| Urbanity | 5.0715 | 0.5155 | 0.9671 | 0.4006 | 80.9% | 22.3% |
|  | (3.5618) | (1.7434) | (1.7385) | (1.0738) |  |  |

Note: Robust standard errors in parentheses. **, * statistically significant at .01, .05 level.

**Results for higher age threshold of 75**

*Table 4: Reduction in Strength of Spatial Patterns in Observed Premature Mortality Versus Spatial Patterns in Residuals from the Socioeconomic Empirical Model*

|  | Observed | Observed | Residuals | Residuals | Decline | Decline |
| --- | --- | --- | --- | --- | --- | --- |
|  | male | Female | male | female | male | female |
| Northness | 0.0098** | 0.0083** | 0.0004 | 0.0006 | 95.9% | 92.8% |
|  | (0.0015) | (0.0011) | (0.0005) | (0.0004) |  |  |
| Westness | 0.0085** | 0.0057** | 0.0022* | 0.0013 | 74.1% | 77.2% |
|  | (0.0022) | (0.0016) | (0.0009) | (0.0007) |  |  |
| Centrality | 0.0130** | 0.0101** | 0.0011 | 0.0008 | 91.5% | 92.1% |
|  | (0.0016) | (0.0012) | (0.0006) | (0.0004) |  |  |
| Contiguity | 0.6951** | 0.7782** | 0.0502 | 0.1030** | 92.8% | 86.8% |
|  | (0.0749) | (0.0615) | (0.0295) | (0.0272) |  |  |
| Proximity | 1.8500** | 1.9617** | -0.1291 | 0.0083 | 107.0% | 99.6% |
|  | (0.4274) | (0.4322) | (0.1472) | (0.1565) |  |  |
| Urbanity | 33.7498* | 10.5218 | 3.2746 | 1.4596 | 90.3% | 86.1% |
|  | (13.6581) | (8.3259) | (3.2891) | (2.1770) |  |  |

Note: Robust standard errors in parentheses. **, * statistically significant at .01, .05 level.

**Results for observations weighted by population size**

*Table 4: Reduction in Strength of Spatial Patterns in Observed Premature Mortality Versus Spatial Patterns in Residuals from the Socioeconomic Empirical Model*

|  | Observed | Observed | Residuals | Residuals | Decline | Decline |
| --- | --- | --- | --- | --- | --- | --- |
|  | male | Female | male | female | male | female |
| Northness | 0.0087** | 0.0065** | -0.0002 | 0.0001 | 102.3% | 98.5% |
|  | (0.0014) | (0.0009) | (0.0006) | (0.0004) |  |  |
| Westness | 0.0080** | 0.0053** | 0.0025** | 0.0013* | 68.8% | 75.5% |
|  | (0.0020) | (0.0012) | (0.0008) | (0.0006) |  |  |
| Centrality | 0.0115** | 0.0083** | 0.0007 | 0.0005 | 93.9% | 94.0% |
|  | (0.0018) | (0.0011) | (0.0007) | (0.0005) |  |  |
| Contiguity | 0.7787** | 0.8080** | 0.0362 | 0.0900* | 95.4% | 88.9% |
|  | (0.1014) | (0.0883) | (0.0425) | (0.0421) |  |  |
| Proximity | 2.4248** | 2.4767** | 0.0479 | 0.2311 | 98.0% | 90.7% |
|  | (0.5436) | (0.5196) | (0.1859) | (0.1866) |  |  |
| Urbanity | 9.2923 | -1.0306 | 1.5425 | 0.8264 | 83.4% | 180.2%%) |
|  | (9.6307) | (5.1288) | (1.8779) | (1.2682) |  |  |

Note: Robust standard errors in parentheses. **, * statistically significant at .01, .05 level.
