# Supplementary material for: Regional inequalities in premature mortality in Great Britain
Source: PLoS One. 2018 Feb 28;13(2):e0193488. doi: 10.1371/journal.pone.0193488 (PMC5831001; doi:10.1371/journal.pone.0193488)
Supplement: S1 Table — (DOCX) [file pone.0193488.s002.docx]

**S1 Table. Results for lower age threshold of 60 of Table 4** Reduction in Strength of Spatial Patterns in Observed Premature Mortality Versus Spatial Patterns in Residuals from the Socioeconomic Empirical Model.

|  | Observed | Observed | Residuals | Residuals | Decline | Decline |
| --- | --- | --- | --- | --- | --- | --- |
|  | male | Female | male | female | male | female |
| Northness | 0.0043** | 0.0026** | 0.0003 | 0.0002 | 93.0% | 92.3% |
|  | (0.0005) | (0.0003) | (0.0003) | (0.0002) |  |  |
| Westness | 0.0048** | 0.0023** | 0.0007 | 0.0002 | 85.4% | 91.3% |
|  | (0.0009) | (0.0005) | (0.0004) | (0.0003) |  |  |
| Centrality | 0.0065** | 0.0036** | 0.0006 | 0.0003 | 90.8% | 91.7% |
|  | (0.0006) | (0.0003) | (0.0003) | (0.0002) |  |  |
| Contiguity | 0.7316** | 0.7221** | 0.0207 | 0.0360 | 97.2% | 95.0% |
|  | (0.0805) | (0.0722) | (0.0345) | (0.0383) |  |  |
| Proximity | 2.0885** | 1.9590** | 0.0206 | 0.1480 | 99.0% | 92.4% |
|  | (0.4501) | (0.4179) | (0.2024) | (0.2011) |  |  |
| Urbanity | 5.0715 | 0.5155 | 0.9671 | 0.4006 | 80.9% | 22.3% |
|  | (3.5618) | (1.7434) | (1.7385) | (1.0738) |  |  |

Note: Robust standard errors in parentheses. **, * statistically significant at .01, .05 level.
