# Supplementary material for: Regional inequalities in premature mortality in Great Britain
Source: PLoS One. 2018 Feb 28;13(2):e0193488. doi: 10.1371/journal.pone.0193488 (PMC5831001; doi:10.1371/journal.pone.0193488)
Supplement: S2 Table — Reduction in Strength of Spatial Patterns in Observed Premature Mortality Versus Spatial Patterns in Residuals from the Socioeconomic Empirical Model. (DOCX) [file pone.0193488.s003.docx]

**S2 Table. Results for higher age threshold of 75 of Table 4**. Reduction in Strength of Spatial Patterns in Observed Premature Mortality Versus Spatial Patterns in Residuals from the Socioeconomic Empirical Model.

|  | Observed | Observed | Residuals | Residuals | Decline | Decline |
| --- | --- | --- | --- | --- | --- | --- |
|  | male | Female | male | female | male | female |
| Northness | 0.0098** | 0.0083** | 0.0004 | 0.0006 | 95.9% | 92.8% |
|  | (0.0015) | (0.0011) | (0.0005) | (0.0004) |  |  |
| Westness | 0.0085** | 0.0057** | 0.0022* | 0.0013 | 74.1% | 77.2% |
|  | (0.0022) | (0.0016) | (0.0009) | (0.0007) |  |  |
| Centrality | 0.0130** | 0.0101** | 0.0011 | 0.0008 | 91.5% | 92.1% |
|  | (0.0016) | (0.0012) | (0.0006) | (0.0004) |  |  |
| Contiguity | 0.6951** | 0.7782** | 0.0502 | 0.1030** | 92.8% | 86.8% |
|  | (0.0749) | (0.0615) | (0.0295) | (0.0272) |  |  |
| Proximity | 1.8500** | 1.9617** | -0.1291 | 0.0083 | 107.0% | 99.6% |
|  | (0.4274) | (0.4322) | (0.1472) | (0.1565) |  |  |
| Urbanity | 33.7498* | 10.5218 | 3.2746 | 1.4596 | 90.3% | 86.1% |
|  | (13.6581) | (8.3259) | (3.2891) | (2.1770) |  |  |

Note: Robust standard errors in parentheses. **, * statistically significant at .01, .05 level.
