# Supplementary material for: Regional inequalities in premature mortality in Great Britain
Source: PLoS One. 2018 Feb 28;13(2):e0193488. doi: 10.1371/journal.pone.0193488 (PMC5831001; doi:10.1371/journal.pone.0193488)
Supplement: S3 Table — Variables employed in spatial pattern recognition (for Great Britain) (Tables 1 and 3 in text). (DOCX) [file pone.0193488.s004.docx]

**S3 Table. Summary of descriptive variable information.** Variables employed in spatial pattern recognition (for Great Britain) (Tables 1 and 3 in text).

| Variable | N | mean | s.d. | min | max |
| --- | --- | --- | --- | --- | --- |
| Male premature mortality rate | 378 | 18790.26 | 3498.20 | 12555.0 | 33250.36 |
| Female premature mortality rate | 378 | 12699.00 | 2280.96 | 8445.00 | 21412.44 |
| Northness | 378 | 303858.1 | 176933.7 | 54268.21 | 1168120 |
| Westness | 378 | -436517 | 101156.7 | -647805 | -111367 |
| Centrality | 378 | 197654.7 | 163328.7 | 3986.234 | 991106 |
| Contiguity (male) | 373 | 18114.08 | 2543.154 | 12555 | 27422.52 |
| Contiguity (female) | 373 | 12306.95 | 1756.166 | 7639 | 18891.13 |
| Proximity (male) | 378 | 18815.57 | 452.5231 | 18072.55 | 20138.17 |
| Proximity (female) | 378 | 18815.57 | 452.5231 | 18072.55 | 20138.17 |
| Urbanity | 378 | 15.58807 | 29.26909 | 0.09 | 321.1 |

Note: 5 local authorities are not contiguous to any other authorities and therefore have missing values for the contiguity variable.
