# Supplementary material for: Regional inequalities in premature mortality in Great Britain
Source: PLoS One. 2018 Feb 28;13(2):e0193488. doi: 10.1371/journal.pone.0193488 (PMC5831001; doi:10.1371/journal.pone.0193488)
Supplement: S4 Table — Variables employed in regressing premature mortality on its socioeconomic determinants (Table 2). (DOCX) [file pone.0193488.s005.docx]

**S4 Table. Summary of descriptive variable information.** Variables employed in regressing premature mortality on its socioeconomic determinants (Table 2).

| Variable | | N | | mean | s.d. | | min | max | |
| --- | --- | --- | --- | --- | --- | --- | --- | --- | --- |
| Male premature mortality rate | | 378 | | 0.188 | 0.035 | | 0.125 | 0.333 | |
| Female premature mortality rate | | 378 | | 0.127 | 0.023 | | 0.845 | 0.214 | |
| Mean income | | 378 | | 33572.15 | 8599.59 | | 22338.00 | 119645.00 | |
| Benefit claimants | | 378 | | 2.634 | 1.291 | | 0.800 | 7.100 | |
| Highest educational qualification composition | |  | | |  | | |  |  |
| GCSE (grades D-G) | | 378 | | 0.144 | 0.035 | | 0.058 | 0.283 | |
| GCSE (grades A-C) | | 378 | | 0.156 | 0.019 | | 0.078 | 0.186 | |
| A level | | 378 | | 0.120 | 0.017 | | 0.076 | 0.192 | |
| Certificate of higher education and above | | 378 | | 0.268 | 0.074 | | 0.142 | 0.536 | |
| Employment by economic sector composition | |  | | |  | | |  |  |
| Agriculture | | 378 | | 0.012 | 0.016 | | 0.000 | 0.100 | |
| Mining | | 378 | | 0.003 | 0.007 | | 0.000 | 0.089 | |
| Manufacturing | | 378 | | 0.094 | 0.039 | | 0.018 | 0.237 | |
| Gas & Electricity | | 378 | | 0.006 | 0.004 | | 0.001 | 0.039 | |
| Water | | 378 | | 0.007 | 0.003 | | 0.001 | 0.031 | |
| Construction | | 378 | | 0.080 | 0.015 | | 0.029 | 0.125 | |
| Retail | | 378 | | 0.160 | 0.021 | | 0.095 | 0.235 | |
| Transport | | 378 | | 0.049 | 0.017 | | 0.024 | 0.155 | |
| Hospitality | | 378 | | 0.056 | 0.017 | | 0.032 | 0.145 | |
| Information Technology | | 378 | | 0.036 | 0.021 | | 0.010 | 0.126 | |
| Finance | | 378 | | 0.040 | 0.025 | | 0.007 | 0.215 | |
| Real estate | | 378 | | 0.014 | 0.004 | | 0.006 | 0.034 | |
| Academic/Science | | 378 | | 0.062 | 0.027 | | 0.020 | 0.192 | |
| Administration | | 378 | | 0.047 | 0.009 | | 0.027 | 0.088 | |
| Public Administration | | 378 | | 0.062 | 0.021 | | 0.023 | 0.260 | |
| Education | | 378 | | 0.097 | 0.018 | | 0.059 | 0.236 | |
| Health | | 378 | | 0.127 | 0.023 | | 0.067 | 0.191 | |
| Socioeconomic status composition | |  | | |  | | |  |  |
| Higher managerial | | 378 | | 0.099 | 0.035 | | 0.039 | 0.215 | |
| Lower managerial | | 378 | | 0.210 | 0.032 | | 0.128 | 0.320 | |
| Intermediate occupations | | 378 | | 0.132 | 0.019 | | 0.079 | 0.196 | |
| Small employers | | 378 | | 0.098 | 0.026 | | 0.049 | 0.185 | |
| Lower supervisory | | 378 | | 0.075 | 0.015 | | 0.030 | 0.117 | |
| Semi-routine occupations | | 378 | | 0.146 | 0.027 | | 0.069 | 0.220 | |
| Routine occupations | | 378 | | 0.117 | 0.034 | | 0.043 | 0.229 | |
| Ethnic composition | |  | | |  | | |  |  |
| Mixed | | 378 | | 0.017 | 0.014 | | 0.002 | 0.076 | |
| Asian | | 378 | | 0.052 | 0.073 | | 0.004 | 0.435 | |
| Black | | 378 | | 0.021 | 0.042 | | 0.000 | 0.272 | |
| White | | 378 | | 0.904 | 0.123 | | 0.290 | 0.993 | |

Note: in table 2, premature mortality is expressed as a percentage.
