# Supplementary material for: Regional inequalities in premature mortality in Great Britain
Source: PLoS One. 2018 Feb 28;13(2):e0193488. doi: 10.1371/journal.pone.0193488 (PMC5831001; doi:10.1371/journal.pone.0193488)
Supplement: S5 Table — Reduction in Strength of Spatial Patterns in Observed Premature Mortality Versus Spatial Patterns in Residuals from the Socioeconomic Empirical Model. (DOCX) [file pone.0193488.s006.docx]

**S5 Table. Results for observations weighted by population size of Table 4.** Reduction in Strength of Spatial Patterns in Observed Premature Mortality Versus Spatial Patterns in Residuals from the Socioeconomic Empirical Model.

|  | Observed | Observed | Residuals | Residuals | Decline | Decline |
| --- | --- | --- | --- | --- | --- | --- |
|  | male | Female | male | female | male | female |
| Northness | 0.0087** | 0.0065** | -0.0002 | 0.0001 | 102.3% | 98.5% |
|  | (0.0014) | (0.0009) | (0.0006) | (0.0004) |  |  |
| Westness | 0.0080** | 0.0053** | 0.0025** | 0.0013* | 68.8% | 75.5% |
|  | (0.0020) | (0.0012) | (0.0008) | (0.0006) |  |  |
| Centrality | 0.0115** | 0.0083** | 0.0007 | 0.0005 | 93.9% | 94.0% |
|  | (0.0018) | (0.0011) | (0.0007) | (0.0005) |  |  |
| Contiguity | 0.7787** | 0.8080** | 0.0362 | 0.0900* | 95.4% | 88.9% |
|  | (0.1014) | (0.0883) | (0.0425) | (0.0421) |  |  |
| Proximity | 2.4248** | 2.4767** | 0.0479 | 0.2311 | 98.0% | 90.7% |
|  | (0.5436) | (0.5196) | (0.1859) | (0.1866) |  |  |
| Urbanity | 9.2923 | -1.0306 | 1.5425 | 0.8264 | 83.4% | 180.2%%) |
|  | (9.6307) | (5.1288) | (1.8779) | (1.2682) |  |  |

Note: Robust standard errors in parentheses. **, * statistically significant at .01, .05 level.
