# Supplementary material for: Regional inequalities in premature mortality in Great Britain
Source: PLoS One. 2018 Feb 28;13(2):e0193488. doi: 10.1371/journal.pone.0193488 (PMC5831001; doi:10.1371/journal.pone.0193488)
Supplement: S6 Table — Reduction in Strength of Spatial Patterns in Observed Premature Mortality Versus Spatial Patterns in Residuals from the Socioeconomic Empirical Model. (DOCX) [file pone.0193488.s007.docx]

**S6 Table. Results from outlier analysis of table 4.** Reduction in Strength of Spatial Patterns in Observed Premature Mortality Versus Spatial Patterns in Residuals from the Socioeconomic Empirical Model.

|  | Observed | Observedd | Residuals | Residuals | Decline | Decline |
| --- | --- | --- | --- | --- | --- | --- |
|  | male | Female | male | female | male | female |
| Northness | 0.0091** | 0.0064** | 0.0001 | 0.0000 | 98.9% | 100.0% |
|  | (0.0009) | (0.0006) | (0.0005) | (0.0004) |  |  |
| Westness | 0.0053** | 0.0044** | 0.0014* | 0.0011* | 73.6% | 75.0% |
|  | (0.0014) | (0.0009) | (0.0007) | (0.0005) |  |  |
| Centrality | 0.0119** | 0.0082** | 0.0005 | 0.0002 | 95.8% | 97.6% |
|  | (0.0009) | (0.0006) | (0.0006) | (0.0004) |  |  |
| Contiguity | 0.7772** | 0.8344** | 0.0215 | 0.0589 | 97.2% | 92.9% |
|  | (0.0546) | (0.0462) | (0.0325) | (0.0315) |  |  |
| Proximity | 1.8575** | 1.5939** | -0.2066 | -0.0669 | 111.1% | 104.2% |
|  | (0.3422) | (0.3335) | (0.1485) | (0.1619) |  |  |
| Urbanity | 42.4830** | 14.8333** | 0.4785 | -1.5115 | 98.9% | 110.2% |
|  | (7.3797) | (4.6325) | (2.6025) | (1.9408) |  |  |

Note: Robust standard errors in parentheses. **, * statistically significant at .01, .05 level.
